# A-to-I editing in the miRNA seed region regulates target mRNA selection and silencing efficiency

**DOI:** 10.1093/nar/gku662

**Published:** 2014-07-23

**Authors:** Hideaki Kume, Kimihiro Hino, Josephine Galipon, Kumiko Ui-Tei

**Affiliations:** 1 Department of Biological Sciences, Graduate School of Science, The University of Tokyo, 7-3-1 Hongo, Bunkyo-ku, Tokyo 113-0033, Japan; 2 Faculty of Medicine, The University of Tokyo, 7-3-1 Hongo, Bunkyo-ku, Tokyo 113-0033, Japan

## Abstract

Hydrolytic deamination of adenosine to inosine (A-to-I) by adenosine deaminases acting on RNA (ADARs) is a post-transcriptional modification which results in a discrepancy between genomic DNA and the transcribed RNA sequence, thus contributing to the diversity of the transcriptome. Inosine preferentially base pairs with cytidine, meaning that A-to-I modifications in the mRNA sequences may be observed as A-to-G substitutions by the protein-coding machinery. Genome-wide studies have revealed that the majority of editing events occur in non-coding RNA sequences, but little is known about their functional meaning. MiRNAs are small non-coding RNAs that regulate the expression of target mRNAs with complementarities to their seed region. Here, we confirm that A-to-I editing in the miRNA seed duplex globally reassigns their target mRNAs *in vivo*, and reveal that miRNA containing inosine in the seed region exhibits a different degree of silencing efficiency compared to the corresponding miRNA with guanosine at the same position. The difference in base-pairing stability, deduced by melting temperature measurements, between seed-target duplexes containing either C:G or I:C pairs may account for the observed silencing efficiency. These findings unequivocally show that C:G and I:C pairs are biologically different in terms of gene expression regulation by miRNAs.

## INTRODUCTION

Adenosine-to-inosine (A-to-I) RNA editing is the site-specific hydrolytic deamination of A-to-I by adenosine deaminases acting on RNAs (ADARs), which occurs within mainly double-stranded RNA regions. Because inosine residues preferentially base pair with cytidines, inosine residues in the mRNA sequences are recognized as guanosines by the translational machinery in eukaryotes, occasionally leading to the alteration of codons. Famous examples of editing within mRNA coding sequences include neurotransmitter receptor or ion channel mRNAs, such as the 2-amino-3-(3-hydroxy-5-methyl-isoxazol-4-yl) propanoic acid (AMPA) receptor subunit, the glutamate receptor (GluR)-B (1), the serotonine-2C receptor (2), the Kv1.1 voltage-dependent potassium channel (3) and the γ-aminobutyric acid (GABA) A receptor subunit alpha3 mRNAs (4). However, only a limited number of protein-coding genes is subjected to A-to-I editing; indeed, the majority of A-to-I editing sites have been identified within non-coding regions of the transcriptome (5,6).

MicroRNAs (miRNAs) are evolutionarily conserved single-stranded non-coding RNAs of 20–22 nucleotides (nts) in length (7). They suppress the expression of protein-coding genes via partial nucleotide sequence complementarity, and play important roles in a broad range of biological processes including development, cellular differentiation, proliferation, apoptosis and the pathogenesis of human diseases such as cancer and metabolic disorders (8). In human, more than 1800 miRNAs have been identified so far (9). In canonical human miRNA biogenesis, primary miRNA transcripts (pri-miRNAs) are initially transcribed forming stem-loop structures. Maturation of miRNAs occurs in two steps. First, pri-miRNAs are processed in the nucleus to ∼70 nt hairpins with a 2 nt 3′ overhang referred to as precursor miRNAs (pre-miRNAs) by Drosha (10) associated with the microprocessor DGCR8 (11). In the cytoplasm, they are further cleaved by Dicer to yield approximately 22 nt-long miRNA duplexes. The miRNA duplexes are then loaded onto the Argonaute (AGO) protein within the RNA-induced silencing complex (RISC), and unwound into single-stranded mature miRNAs. In this process, the RNA strand with relatively relaxed structure, such as internal bulges or mismatches, in the 5′ terminal duplex is preferentially entrapped on the RISC (12). The mature miRNA then guides RISC to target mRNA by partial sequence complementarity mainly in the seed sequence (nucleotide positions 2–8 from the 5′ end of the miRNA) (13,14).

RNA interference (RNAi), which is extensively used for intended gene silencing, requires a near perfect sequence match between the guide strand of small interfering RNA (siRNA) and target mRNA, has been used to study gene function in a variety of organisms and it holds great promise for therapeutic applications. The seed region (nucleotides 2–8 from the 5′-end) of the siRNA guide strand is known to downregulate the expression of genes outside of the canonical targets with weak interaction due to the seed sequence, which is commonly called ‘off-target’ genes. Thus, the mechanism of the off-target effect due to siRNA is considered to be very similar, if not identical, to that of microRNA-based gene silencing.

Our previous study revealed that the capability of siRNA to induce off-target effects is highly correlated with its calculated melting temperature (Tm) and the free energy change (ΔG) for formation of the protein-free seed-target duplex (15), indicating that thermodynamic stability of the RNA duplex formed between the seed and the target is one of the major factors in determining the degree of off-target effects: the highly stable seed-target duplex function as a positive silencing regulator but the unstable duplex is a negative regulator. However, unlike siRNA off-target effects, the efficiency of miRNA-mediated gene silencing was not simply correlated with the stability in the seed-target duplex. It was previously shown that the efficacy of miRNA-mediated gene silencing was partly determined by combined thermodynamic parameters of both the 5′-terminal 5-bp duplex and the seed-target duplex (12). The stability in the 5′ terminal end is known to regulate the efficiency of small RNA loading on the RISC: an RNA strand with a thermodynamically less stable 5′ terminal is preferentially entrapped on the RISC compared to the RNA strand with a stable 5′ terminal (16–18). The seed region (nucleotides 2–8) actually overlaps with the 5′-end (nucleotides 1–5). These two regions were considered to function coordinately but independently, because the stability in the seed-target duplex is defined by the nucleotide sequence, whereas the stability in the 5′-terminal duplex is attributable to structural features as well as nucleotide sequence (12).

The first instance of miRNA A-to-I editing was detected by polymerase chain reaction (PCR) amplification and sequencing of the pri-miR-22 region (19). Subsequently, a systematic survey of miRNA editing focused on approximately 200 pri-miRNAs (20). In the past few years, deep sequencing of small RNAs has allowed the identification of A-to-I editing sites in the miRNA of a variety of organisms, cell lines and tissues (21–27), revealing not only pri-miRNAs but also a number of pre-miRNAs as ADAR targets. The editing of miRNAs has the potential to exert a profound effect on their biogenesis, such as inhibition—and sometimes promotion—of Drosha or Dicer processing (20,28,29). A-to-I editing may induce degradation of miRNAs by the Tudor-SN inosine-specific endonuclease (28), and was also shown to regulate strand selection during RISC loading (30). Furthermore, when the edited site is positioned in miR-376a-5p seed region, the target genes are shown to shift from those containing complementary sequences with the non-edited A-containing seed sequence to those with the G-containing seed sequence (31). Thus, as is observed in mRNA coding sequences, the conversion from A-to-I has been considered to be equivalent to an adenosine to guanosine change. However, it has long been known that I:C forms a thermodynamically weaker base pair compared to G:C, and that A-to-I editing induces a change in nucleic acid geometry. Consistent with such physicochemical characteristics, our study reveals that I:C and G:C base-pairing between miRNA and their targets do not contribute equally to miRNA-mediated downregulation of gene expression, and melting curves showed that I:C pairs are slightly less stable than G:C pairs. We provide evidence that edited miRNAs with an inosine in the 5′-terminal and seed region may exhibit different degrees of *in vivo* silencing activity compared to those with guanosines at the same positions.

## MATERIALS AND METHODS

### Preparation of chemically synthesized miRNA duplexes and seed duplexes

**Figure 1.**
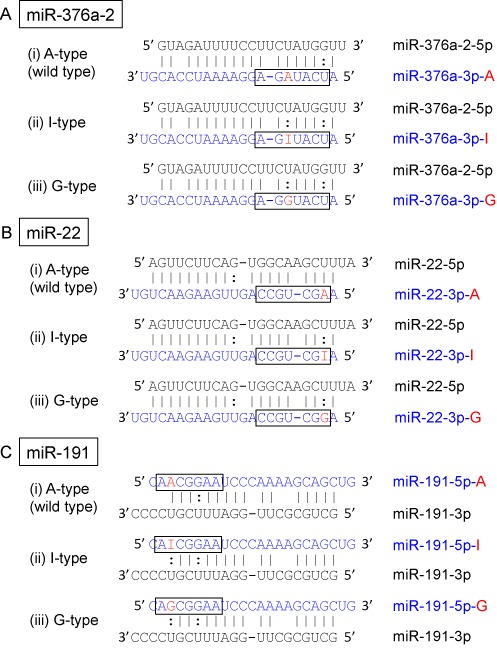
Structures of miRNA duplexes used in this study. (**A**) Three types of miR-376a-2 duplex and its derivatives. A-type miR-376a-2 duplex is the wild-type duplex formed between miR-376a-2–5p and miR-376a-3p-A, in which the possible editing site of adenosine is situated at position +6 from the 5′ end. I-type or G-type miR-376a-2 duplex is composed of miR-376a-2–5p and miR-376a-3p-I or miR-376a-3p-G. (**B**) Three types of miR-22 duplex and its derivatives. A-type miR-22 duplex is the wild-type duplex formed between miR-22–5p and miR-22–3p-A, in which the possible editing site of adenosine is situated at position +2. I-type or G-type miR-22 duplex is composed of miR-22–5p and miR-22–3p-I or miR-22–3p-G. (**C**) Three types of miR-191 duplex and its derivatives. A-type miR-191 duplex is the wild-type duplex formed between miR-191–3p and miR-191–5p-A, in which adenosine is situated at position +3. I-type or G-type miR-191 duplexes are composed of miR-191–3p and either miR-191–5p-I or miR-191–5p-G, respectively.

RNA oligonucleotides corresponding to the 5p and 3p strands of mature miRNA duplexes (21–24-nt in length) and 7-mer oligonucleotides corresponding to the mRNA binding sequence complementary to the active seed (nucleotides 2–8) were chemically synthesized (Sigma or Genepharma) in accordance with the sequences registered in the miRBase and annealed to form either 5p:3p mature miRNA duplexes and seed:mRNA binding site duplexes (9). In addition, the same oligonucleotides containing either inosine (I-type) or guanosine (G-type) instead of adenosine (A-type) at the possible editing sites were also synthesized. The miRNA strand with wild-type adenosine, inosine and guanosine at the editing site are referred to as miRNA-A, -I and -G, respectively. Both strands of miRNAs or seed duplexes were mixed to a 1:1 ratio in a solution of 10 mM NaCl and 20 mM Tris–HCl (pH 7.5), and annealed by incubation at 95ºC for 5 min followed by cooling down to room temperature. The annealed miRNA duplexes containing either adenosine, inosine or guanosine at the editing sites are referred to as A-type, I-type and G-type miRNAs, respectively. The sequence of the synthetic mature miRNAs (miR-376a-2, miR-22, miR-191) and their structures are shown in Figure 1, those of the seed RNA duplexes are shown in Figure 4. siGY441 with a sequence unrelated to the *Renilla* luciferase gene was used as a negative control. Duplex formation was verified by electrophoresis on a 15% polyacrylamide gel in Tris Borate EDTA (TBE) buffer.

**Figure 2.**
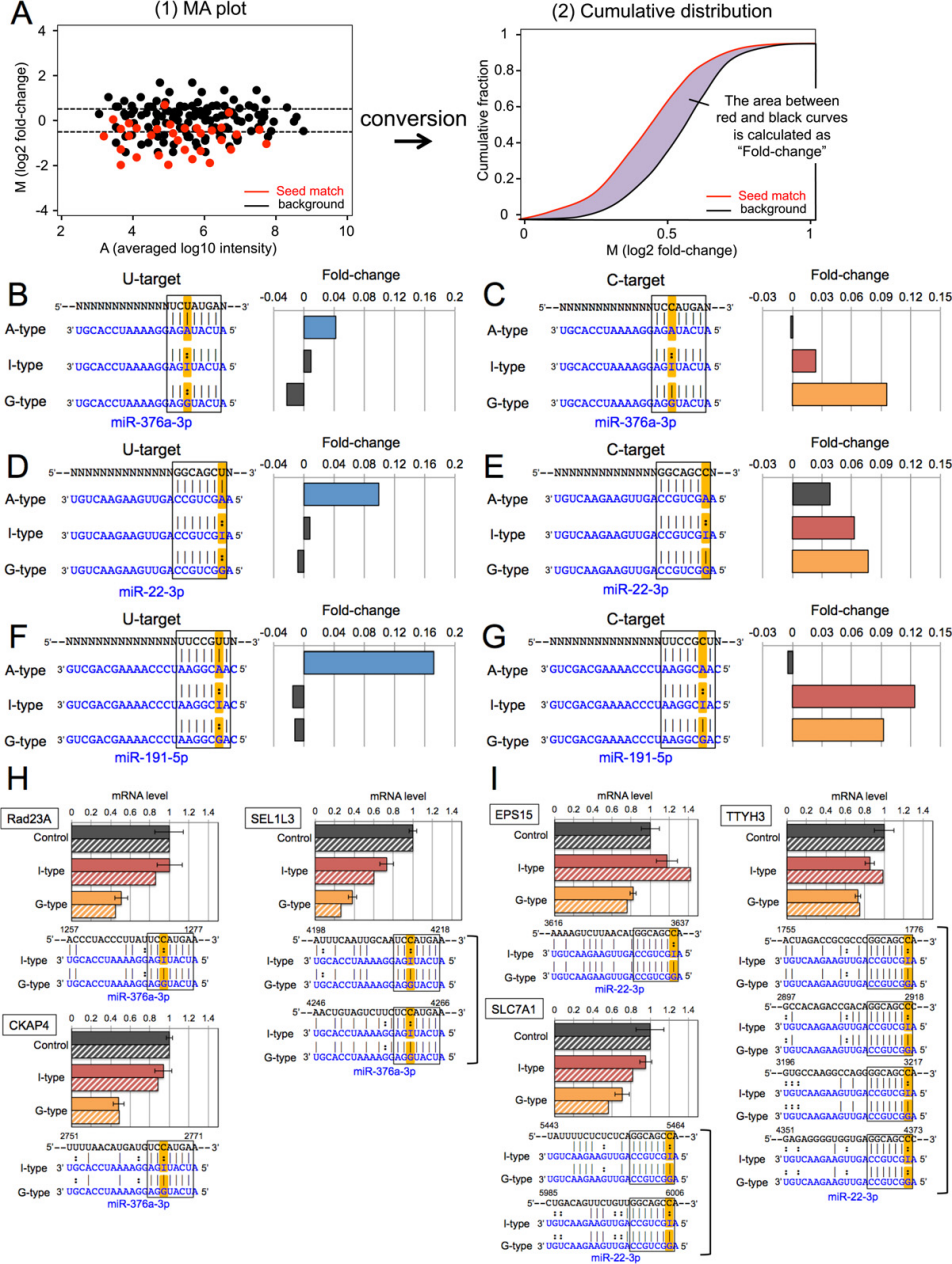
Microarray and real-time PCR analysis of the expression levels of target transcripts with seed complementary sequences. (**A**) Schematic model describing the analysis workflow for microarray data shown in Supplementary Figures S1–S3. (1) MA plot: the vertical bar indicates the mean log2 of signal intensities relative to those of mock transfection (M value), and the horizontal bar indicates the averaged log10 signal intensities of mock and miRNA transfections (A value). The red plots show seed-matched transcripts and the black plots show background transcripts. (2) Cumulative distribution: the horizontal axis indicates the ‘M value’, and the vertical axis is the cumulative fraction of mRNAs. The red line indicates the cumulative curve of seed-matched transcripts, and the the black line that of background transcripts. The gray area indicates the fold-change in the expression of seed-matched transcripts compared to those of background transcripts. Each of miR376a-2 (**B**) and (**C**), miR-22 (**D**) and (**E**) and miR-191 (**F**) and (**G**) duplexes were transfected into HeLa cells, and the changes in expression levels of transcripts with U-target (B), (D), (F) or C-target (C), (E), (G) sequences were analyzed by microarray. The complementarity between seed region and target mRNAs were shown in left panel in A–F. In the target sequence, N indicates any given nucleotide. In each experiment, three types of miRNAs (A-type, I-type, G-type) was respectively used. Fold-change indicates the difference of gene expression levels between mean log2 of signal intensities of each miRNA targets and background genes. The expression levels of three target mRNAs of miR-376a-3p (Rad23A, CKAP4 and SEL1L3) (H) or miR-22–3p (EPS15, SLC7A1 and TTYH3) (I) by mock transfection (black bar), I-type miRNA (red bar), or G-type miRNA (yellow bar) were measured by qRT-PCR, and compared with the microarray data (each of black, red, or yellow diagonal bar). Note that the number of seed-complementary target sites ranged from one to four. The seed regions and their complementary site are surrounded by a box, and the possible editing sites were highlighted in orange. In (**H**) and (**I**), positions relative to the 5′ end of mRNA were shown. In (H) and (I), a bar indicates the standard deviation calculated from three independent experiments.

**Figure 3.**
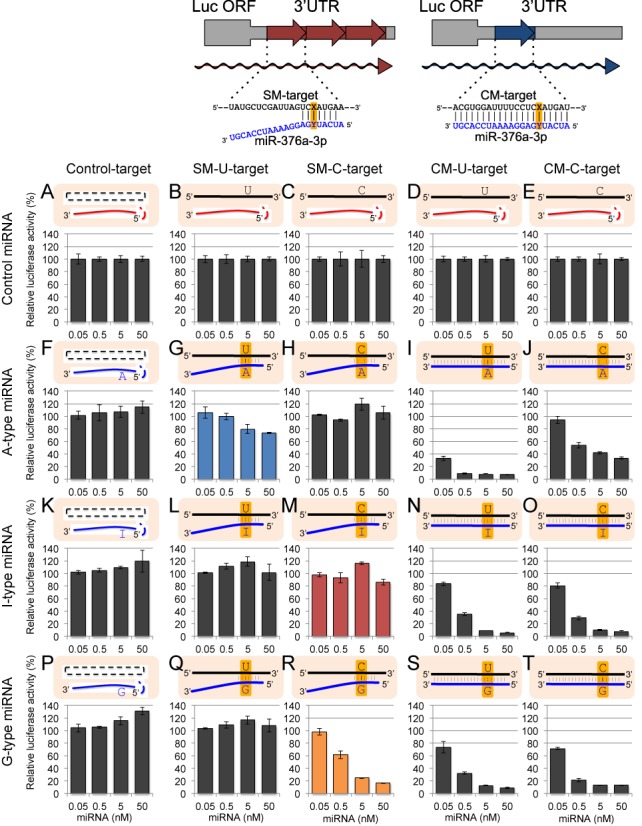
The results of reporter assays using miR-376a-2 duplex and its derivatives. The upper panels indicate the schematic structures of psiCHECK-SM-target and psiCHECK-CM-target constructs containing three tandem repeats of SM-target sequences and a CM-target sequence, respectively, in the 3′ UTR of Renilla luciferase gene of psiCHECK vector. Position Y indicates the possible editing site in the mRNA, and position X in the SM- or CM-target sequence indicates the position opposite to the the possible editing site of miRNA. Control siGY441 (**A**)–(**E**), wild-type A-type (**F**)–(**J**), I-type (**K**)–(**O**), or G-type (**P**)–(**T**) miR-376a-2 duplex was transfected with control psiCHECK-1 (A), (F), (K), (P), psiCHECK-SM-U-target (B), (G), (L), (Q), psiCHECK-SM-C-target (C), (H), (M), (R), psiCHECK-CM-U-target (D), (I), (N), (S), or psiCHECK-CM-C-target (E), (J), (O), (T), and pGL3-Control firefly luciferase expression construct were simultaneously transfected into HeLa cells. Relative luciferase activity was measured one day after transfection. Each miRNA was transfected at 0.05, 0.5, 5 and 50 nM, respectively. Complementarity between transfected miRNA and target construct is shown at top of each figure, and the possible editing sites were highlighted. Bar indicates the standard deviation of triplicated samples.

**Figure 4.**
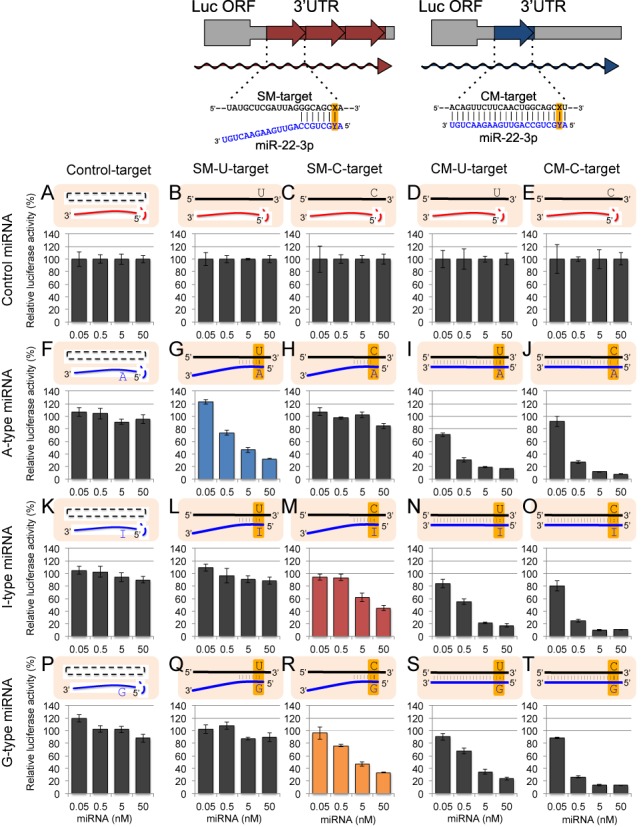
The results of reporter assays using miR-22 duplex and its derivatives. Control siGY441 (**A**)–(**E**), wild-type A-type (**F**)–(**J**), I-type (**K**)–(**O**), or G-type (**P**)–(**T**) miR-22 duplex was transfected with control psiCHECK-1 (A), (F), (K), (P), psiCHECK-SM-U-target (B), (G), (L), (Q), psiCHECK-SM-C-target (C), (H), (M), (R), psiCHECK-CM-U-target (D), (I), (N), (S), or psiCHECK-CM-C-target (E), (J), (O), (T), and pGL3-Control firefly luciferase expression construct were simultaneously transfected into HeLa cells. Other detailed descriptions about this figure are same as those of Figure 3.

### Microarray analysis

Human HeLa cells were cultured in Dulbecco's modified Eagle's medium (DMEM; Gibco BRL) supplemented with 10% heat-inactivated fetal bovine serum (FBS; Mitsubishi Kagaku) at 37°C. The cells inoculated in 12-well plates 3 × 10^5^ cells/ml were transfected with 50 nM of each miRNA duplex using 4 μl of Lipofectamine 2000. At 24 h post-transfection, total RNA was purified with an RNeasy Kit (Qiagen), and RNA quality was assessed using a NanoDrop 2000 spectrophotometer (Thermo Scientific) and a Bioanalyzer (Agilent). cDNA was synthesized from each total RNA sample using an Agilent One Color Spike Mix Kit (Agilent), and used for hybridization to an Agilent Whole Human Genome Microarray (4×44 K multi-pack format) according to the manufacturer's protocol. Data analysis was automated for mock, A-type, I-type and G-type miRNA duplex transfection samples using R code as follows. First, a filter was applied to keep only the spots that were detected with certainty in all four samples. RNA from mock-transfected cells treated with the transfection reagent in the absence of miRNA duplex was used as a control, and the distributions of transcript expression values were normalized across all samples by quantile normalization (32). Agilent chips tend to contain several probes per gene. Since the filter described above selects only the spots for which the signal is not saturated, meaning that the signals are expected to be within the linear range of detection, the signal intensities of plural probes corresponding to a single gene were averaged. Next, we used a Perl script to look up RefSeq genes with one or more regions complementary to the 5p or 3p seed regions (nucleotides 2 to 8) in their 3′ UTR. To enable direct comparison between the seed-matched output and background genes, gene references that were not found in RefSeq were excluded from the microarray data set. Then, the following steps were as follows, in order: (i) discarded the genes which contain a region complementary to the dead seed, which means the seed sequence on the opposite strand of the miRNA containing the editing site; (ii) selected groups of genes with at least one seed-match for either one of four versions of the active seed, containing A, C, G, or U at the potential A-to-I editing site; (iii) eliminated the genes that were included in more than one group, to end up with a list of unique A-, C-, G- and U-seed matches; (iv) output the corresponding lists of genes considered as background: RefSeq genes without dead-seed matches and without either one of the unique A-, C-, G- or U-seed matches, respectively. The fold-change of gene expression level between background and seed-matched genes was obtained by subtracting the respective average log2 of signal intensities of seed-matched target genes from that of background genes for each experiment, which provides an estimate of the area between the red (seed-match) and black (background) curves.

### Quantitative RT-PCR (qRT-PCR)

Total RNA purified from HeLa cells was reverse-transcribed using a High Capacity cDNA Reverse Transcription Kit (Applied Biosystems). The resultant cDNA was subjected to real-time PCR using the Power SYBR Green PCR Master Mix (Applied Biosystems). PCR product levels were monitored and triplicates were analyzed using a StepOnePlus Real-Time PCR system (Applied Biosystems). The expression level of each transcript was first normalized to that of lactate dehydrogenase-A (LDHA), and then to that of the mock transfection control. The PCR primer sets used are listed in Supplementary Table S2.

### Construction of luciferase reporters with complete-matched and seed-matched sequences

The plasmids containing reporter constructs were all derived from psiCHECK-1 (Promega). Oligonucleotides with target sequences completely matched (CM) to each annotated miRNA sequence were chemically synthesized with cohesive *Xho*I/*Eco*RI ends and inserted into psiCHECK-1 at the corresponding restriction sites to generate psiCHECK reporter constructs. The wild-type A-type miRNA target sequence carries an uridine at the position complementary to the adenosine, and are designated as psiCHECK-CM-U-target. psiCHECK reporter constructs with G-type and I-type miRNA target sequences were also constructed, which have a cytidine at the complementary site, and were therefore designated as psiCHECK-CM-C-target. Similarly, three tandem repeats of seed-matched (SM) target sequences, each of which has complementarity with just the 7 nucleotide-long seed sequence, but not with the non-seed region, were also synthesized and named as psiCHECK-SM-U-target or psiCHECK-SM-C-target. The inserted oligonucleotide sequences are shown in Supplementary Table S1. Each of the inserted targets was expressed as part of the 3′-UTR region of *Renilla* luciferase mRNA in the transfected cells.

### Cell culture and RNA silencing activity assay using firefly luciferase reporter system

HeLa cells inoculated in each well of 96-well plates at 2 × 10^4^ cells/100 μl were transfected simultaneously with psiCHECK-CM or –SM-target construct (3.3 ng), pGL3-Control (33 ng; Promega) and miRNA duplex (0.05 nM, 0.5 nM, 5 nM or 50 nM) using 0.67 μl of lipofectamine 2000 per well (Life Technologies). An siRNA against enhanced green fluorescent protein (EGFP) was used as control. The cells were harvested 24 h post-transfection and the relative luciferase activity (*Renilla* luc activity/firefly luc activity) was measured using a Dual-Luciferase Reporter Assay System (Promega). The pGL3-Control encoding firefly luciferase served as a control for the calculation of relative luciferase activity.

### Measurement of Tm values

Both strands of the RNA 7-mer seed duplex were mixed to a 1:1 ratio (final 5 μM) in a solution of 1 M NaCl, 0.5 mM EDTA and 5mM Na_2_HPO_4_ (pH 7.5). Then, the absorbance of samples was monitored at 260 nm from 4 to 95°C at a heating speed of 1°C/min and analyzed using a UV-2550 spectrophotometer with the Thermal Melt Analysis System for Nucleic Acids TMSPC-8 (SHIMADZU). Tm values were calculated by the Two Point Average method (33).

## RESULTS AND DISCUSSION

### Microarray analysis of global conversion of target mRNAs by A-to-I editing in the miRNA seed region

It is well known that miRNA mainly recognizes mRNAs with complementarity to the miRNA seed sequence (nucleotides 2–8) in their 3′-UTRs (13,14). Thus, A-to-I RNA editing in the miRNA seed region is expected to change the set of target mRNAs: wild-type miRNA with adenosine in the seed region recognizes mRNAs with uridines at the complementary position, but the edited form with inosine instead of adenosine in the seed region should target mRNAs with cytidines at the opposite sites. In fact, Kawahara *et al.* (31) reported that miR-376a-5p edited at the +4 site in the seed region silences a different set of genes than its non-edited form.

To monitor the global changes in the identity of downregulated transcripts by miRNAs with or without editing in the seed region, we used three types of miRNAs, miR-376a-2, miR-22 and miR-191. MiR-376a-2 (31) and miR-22 (19) have been reported to have at least one editing site in their respective seed regions, and the editing site in the seed region of miR-191 was suggested by our unpublished results. We prepared three patterns of miRNA duplexes for each of three types of miRNAs (Figure 1): (i) an miRNA duplex was chemically generated using the non-edited wild-type miRNA strand (miR-376a-3p-A, miR-22–3p-A, or miR-191–5p-A) and the wild-type complementary strand (miR-376a-2–5p, miR-22–5p, or miR-191–3p), and named as ‘A-type’ miRNA. (ii) Since adenosine is converted into inosine by ADAR-mediated deamination, the ‘I-type’ miRNA duplex formed between the miRNA strand with inosine at the possible editing site (miR-376a-3p -I, miR-22–3p-I, or miR-191–5p-I) and its wild-type opposite miRNA strand was prepared. (iii) Finally, since an inosine preferentially base pairs with cytidine, similarly to guanosine, the miRNA duplex formed by the miRNA strand with guanosine at the editing site (miR-376a-3p-G, miR-22–3p-G, or miR-191–5p-G) and the wild-type opposite miRNA strand was also prepared, and referred to as ‘G-type’ miRNA. Since all of the miRNA strands without editing (miR-376a-2–5p, miR-22–5p, or miR-191–3p) in the three types of miRNA duplexes contain uridines at the positions opposite to the editing sites, A:U, I:U and G:U pairs are formed at the possible editing positions in the wild-type (A-type), edited type (I-type) and G-type miRNA duplexes, respectively (Figure 1). These miRNA duplexes were respectively transfected into human HeLa cells at 50 nM, and microarray analysis was performed using total RNA extracted 24 h after transfection.

To analyze miRNA-induced global changes in gene expression, mRNAs were divided into four groups based on the presence in their 3′-UTRs of at least one sequence complementary to the seed region containing either A, U, G or C at the possible editing site, respectively. The mRNAs that belonged to more than one of these groups, and those with 3′-UTR complementarity to the dead seed were eliminated to select for targets that may be downregulated by only one type of seed sequence. These separate groups are subsequently referred to as (I) U-target, (II) A-target, (III) C-target, (IV) G-target mRNAs (see Supplementary Figures S1–S3). Consequently, A-type miRNAs are expected to specifically downregulate ‘U-target’ genes, whereas the I-type and G-type miRNAs should downregulate ‘C-target’ genes. The background constitutes of all mRNAs—excluding dead seed targets—that are outside of each individual group. As a result, the background differs slightly between the four groups. The procedure for microarray data analysis is illustrated in Figure 2A. At first, we made an MA plots showing the mean log2 of signal intensities relative to those of mock transfection (M value) and the averaged log10 signal intensities of mock and miRNA transfections (A value), and plotted these values as the vertical and horizontal bars, respectively (Figure 2A(1)). To facilitate understanding, the MA plot was then converted to the cumulative distribution (Figure 2A(2)), in which the horizontal axis indicates the ‘M value’ and the vertical axis is the cumulative fraction of mRNAs. The mean log2 of signal intensities relative to those of mock transfection was calculated for each group of mRNAs and the results are shown as MA plots and cumulative distributions (Supplementary Figures S1–S3). Furthermore, to simplify the results, the ‘area’ between seed-matched mRNAs (red curve in Figure 2A(2)) and background RNAs (black curve in Figure 2A(2)) was calculated and shown as the ‘fold-change’ in Figure 2B–G, with positive values meaning that the target genes are generally more inhibited than background. Furthermore, the Wilcoxon rank-sum test was used to assess whether or not the target genes and background distributions are significantly different (*p* < 1×10^−2^).

As expected, the microarray results for transfection of wild-type (A-type) miR-376a-2 revealed that U-target transcripts, which harbour one or more sequences complementary to the miR-376a-3p seed region UCAUAGA (underline indicating the possible editing site) in their 3′-UTRs, were significantly downregulated. Indeed, the fold-change of expression levels was 0.042 (*p* = 2.14 × 10^−9^), which was highest and significant for U-target transcripts after transfection with the A-type duplex, but not I-type (0.010, *p* = 1.01 × 10^−1^) or G-type (-0.023, *p* = 3.82 × 10^−2^) duplexes (Figure 2B, Supplementary Figure S1). On the other hand, both I-type and G-type miRNA duplexes of miR-376a-2, but not A-type, significantly downregulated C-target genes, by 0.024 (*p* = 1.83 × 10^−3^), 0.096 (*p* = 3.51 × 10^−16^) and -0.002 (*p* = 3.9 × 10^−1^), respectively (Figure 2C, Supplementary Figure S1). These results show that the global inhibitory effect of I-type miR-376a-2 duplex on C-target genes was apparently weaker than that of the G-type duplex. This result clearly suggests that the inosine in the seed region of miR-376a-3p-I (UCAUIGA) did not exert the same extent of inhibition than its guanosine-containing counterpart miR-376a-3p-G (UCAUGGA). In a similar fashion, the wild-type (A-type) miR-22 and miR-191 duplexes significantly repressed the expression levels of U-target mRNAs of miR-22–3p-A and miR-191–3p-A, as indicated by the rather high fold-changes of gene expression of 0.099 (*p* = 1.46 × 10^−23^) and 0.172 (*p* = 2.77 × 10^−24^), respectively which was not the case of I-type and G-type miRNAs (*p* = 3.87∼5.46 × 10^−1^, Figure 2D and E, Supplementary Figures S2 and S3). Similarly, the C-target mRNAs of miR-22–3p-G or miR-191–5p-G were significantly downregulated by the transfection of G-type miR-22 or miR-191 duplexes with fold-changes of gene expression levels of 0.077 (*p* = 9.5 × 10^−25^) and 0.092 (*p* = 3.33 × 10^−5^), respectively (Figure 2E and G, Supplementary Figures S2 and S3). However, the C-target mRNA silencing efficiency exerted by I-type miRNAs differed between miRNA species when compared to the corresponding G-type miRNAs. Although the differences were not as clearly marked as for miR-376a-2, the C-target transcripts seemed to be downregulated strongly by the G-type (0.077, *p* = 9.5 × 10^−25^) than by the I-type (0.063, *p* = 1.68 × 10^−12^) in the case of miR-22, whereas for miR-191, the effect of the I-type (0.124, *p* = 1.25 × 10^−8^) appeared somewhat stronger compared to the G-type (0.092, *p* = 3.33 × 10^−5^) (Figure 2F and G, Supplemental Figures S2 and S3), but the level of significance of these smaller differences are not clear. Nevertheless, these findings suggest that the effect of inosine versus that of guanosine is likely to depend on the miRNA duplex involved and/or the position of the editing site and its sequence context, since nearest neighbor residues may affect the thermodynamics of miRNAs.

The A-type miR-376a-2 and miR-191 duplexes appeared to have little, if any, silencing activity on the predicted C-targets of mature miR-376a-3p or miR-191–5p (Figure 2C and G). On the other hand, the A-type miR-22 duplex induced a slight but significant downregulation of C-target transcripts (0.037, *p* = 1.65 × 10^−5^) (Figure 2E). Since miR-22–3p-A had the largest number of U-targets (688 genes) compared to all other miRNAs tested (152∼501 genes) (Supplemental Figures S1–S3), this may have resulted in non-negligible secondary effects. Alternatively, regions other than the seed might be involved in silencing activity (34).

To confirm the reliability of our microarray data, we selected three genes each from the C-targets of miR-376a-3p and miR-22–3p, respectively, for which the extent of downregulation by I-type and G-type miRNAs was different, and carried out qRT-PCR analysis (Figure 2H and I). In the case of miR-376a-3p, the expression levels of all three mRNAs: Rad23A, Cytoskeleton-associated protein 4 (CKAP4) and sel-1 suppressor of lin-12-like 3 (SEL1L3), appeared to be downregulated weakly by the I-type miR-376a-2 duplex but strongly by the G-type miR-376a-2 duplex in the microarray experiments, which was consistent with qRT-PCR results (Figure 2H). Similarly, for miR-22–3p, the expression of epidermal growth factor receptor pathway substrate 15 (EPS15), solute carrier family 7 (cationic amino acid transporter, y+ system) member 1 (SLC7A1) and tweety family member 3 (TTYH3) appeared weakly repressed by the I-type miR-22 duplex but strongly by the G-type miR-22 duplex both in the microarray experiment and by qRT-PCR (Figure 2I). Thus, the relative transcription levels estimated by qRT-PCR were well correlated with those obtained by the microarray analysis (Figure 2H and I). Based on these results, we conclude that gene silencing efficiency of I-type miRNA is not necessarily the same as that of G-type miRNA.

### Reporter assay of target conversion by A-to-I editing in the miRNA seed region

**Figure 5.**
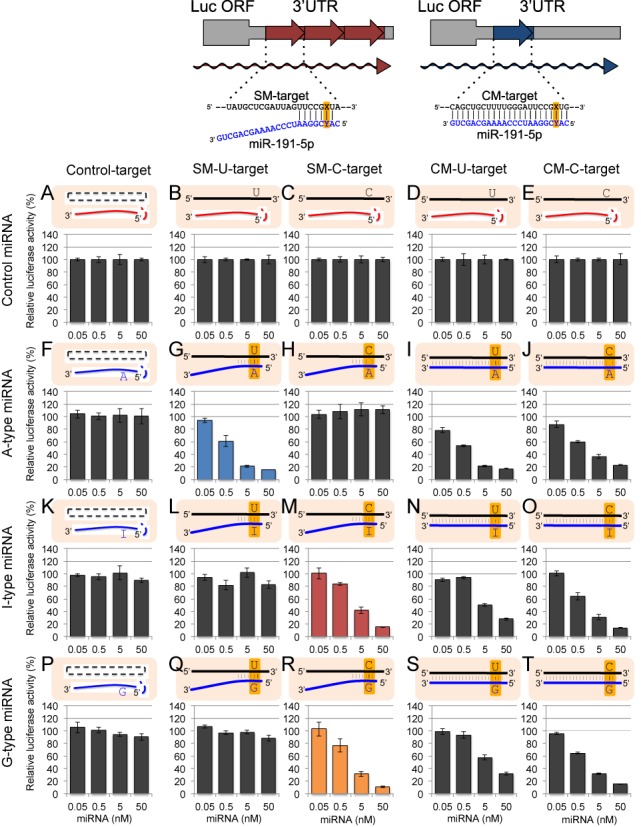
The results of reporter assays using miR-191 duplex and its derivatives. Control siGY441 (**A**)–(**E**), wild-type A-type (**F**)–(**J**), I-type (**K**)–(**O**), or G-type (**P**)–(**T**) miR-191 duplex was transfected with control psiCHECK-1 (A), (F), (K), (P), psiCHECK-SM-U-target (B), (G), (L), (Q), psiCHECK-SM-C-target (C), (H), (M), (R), psiCHECK-CM-U-target (D), (I), (N), (S), or psiCHECK-CM-C-target (E), (J), (O), (T) and pGL3-Control firefly luciferase expression construct were simultaneously transfected into HeLa cells. Other detailed descriptions about this figure are same as those of Figure 3.

**Figure 6.**
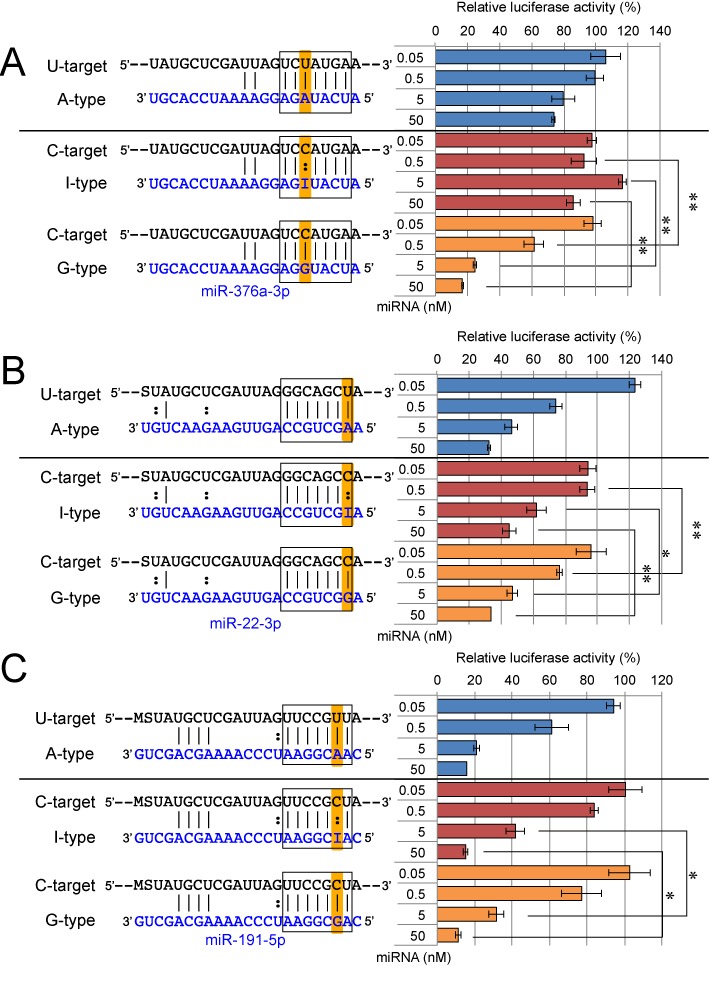
Luciferase reporter assay of silencing efficiencies of A-type, I-type and G-type miRNAs. Wild-type A-type miRNA duplexes [miR-376a-2 (**A**, blue bar), miR-22 (**B**, blue bar), or miR-191 duplex (**C**, blue bar)] and pGL3-Control firefly luciferase expression construct were simultaneously transfected into HeLa cells with each psiCHECK-SM-U-target luciferase reporter construct, and the relative luciferase activity was measured. Both I-type miRNA duplexes [miR-376a-2 (A, red bar), miR-22 (B, red bar), or miR-191 duplex (C, red bar)] and G-type miRNA duplexes [miR-376-a-2 (A, yellow bar), miR-22 (B, yellow bar), or miR-191 duplex (C, yellow bar)] were respectively transfected with pGL3-Control with its psiCHECK-SM-C-target, and relative luciferase activity was measured. Each miRNA was transfected at 0.05, 0.5, 5 and 50 nM, respectively. The seed regions and its complementary site are surrounded by box, and the possible editing sites were highlighted in orange. S indicates cytidine or guanosine, M indicates adenosine or cytidine. (*, 0.05 ≥ *P* > 0.01; **, 0.01 ≥ *P*, Student's *t*-test)

Microarray experiments revealed that the expression of C-target transcripts were downregulated by G-type miRNAs. Moreover, I-type miRNAs could reduce the expression levels of C-target mRNAs, but the reduction levels were not necessarily equivalent to those of G-type miRNAs and the effect of inosine seemed to vary between the miRNA species tested. However, a well-known caveat of microarray experiments is that gene expression may be affected by secondary effects and/or miRNA off-target effects. Therefore, we performed an *in vivo* luciferase assay in order to confirm the direct effect of each miRNA on their target mRNAs. Two types of reporter plasmids were constructed (Figures 3–5): (i) psiCHECK–SM-target, which contains three tandem repeats of seed-matched (SM) sequences with complementarity to the seed region only (Figures 3–5, upper left panels). (ii) psiCHECK–CM-target with a single complete-matched (CM) sequence corresponding to the full-length mature miRNA, as a positive control (Figures 3–5, upper right panels). The SM or CM target sequences were inserted into the 3′-UTR of the *Renilla* luciferase gene in a psiCHECK-1 vector (see Materials and Methods section, upper panels of Figures 3–5). In both constructs, uridine or cytidine was introduced into the target sequence at the opposite position (position X in the target sequences of the upper panels in Figures 3–5) of the possible editing site of miRNA, and shown as SM-U-target or SM-C-target, and CM-U-target or CM-C-target, respectively (Figures 3–5). Each of these reporter constructs was co-transfected into human HeLa cells with pGL3-Control encoding firefly luciferase gene and each miRNA, and relative luciferase activity was measured one day after transfection. The results of luciferase reporter assays for all combinations of targets and miRNAs were shown in Figures 3–5, and those of the specific combinations (SM-U-targets and A-type miRNA, SM-C-targets and I-type miRNAs and SM-C-targets and G-type miRNAs) were picked up and compared in Figure 6. All three A-type wild-type miRNA duplexes (miR-376a-2, miR-22 and miR-191 duplexes) reduced the luciferase activity of their SM-U-targets in a dose-dependent manner, but not that of SM-C-targets (Figures 3–6). Consistently, all of the G-type miRNAs reduced the luciferase activity of their SM-C-targets, but not that of SM-U-targets (Figures 3–6). However, the effects of I-type miRNAs were different between miRNA species: the I-type miR-376a-2 duplex did not significantly repress its SM-C-target (Figures 3 and 6A), whereas the I-type miR-22 (Figures 4 and 6B) and miR-191 duplexes (Figures 5 and 6C) significantly reduced the luciferase activity of SM-C-target reporters, albeit at different levels. These results were well correlated with those of the microarray experiment (Figure 2), with the C-target silencing activity of I-type miRNAs in order of miR-191 > miR-22 > miR-376a-2. Finally, we could verify the effectiveness on CM-targets of all A-type, I-type and G-type miRNA duplexes used in this study, indicating that these miRNAs are functional and not defective (Figures 3–5, CM-target).

### Combinatorial thermodynamic stability in the 5′ terminal duplex and the duplex formed between miRNA seed region and target mRNA may regulate silencing efficiency

**Figure 7.**
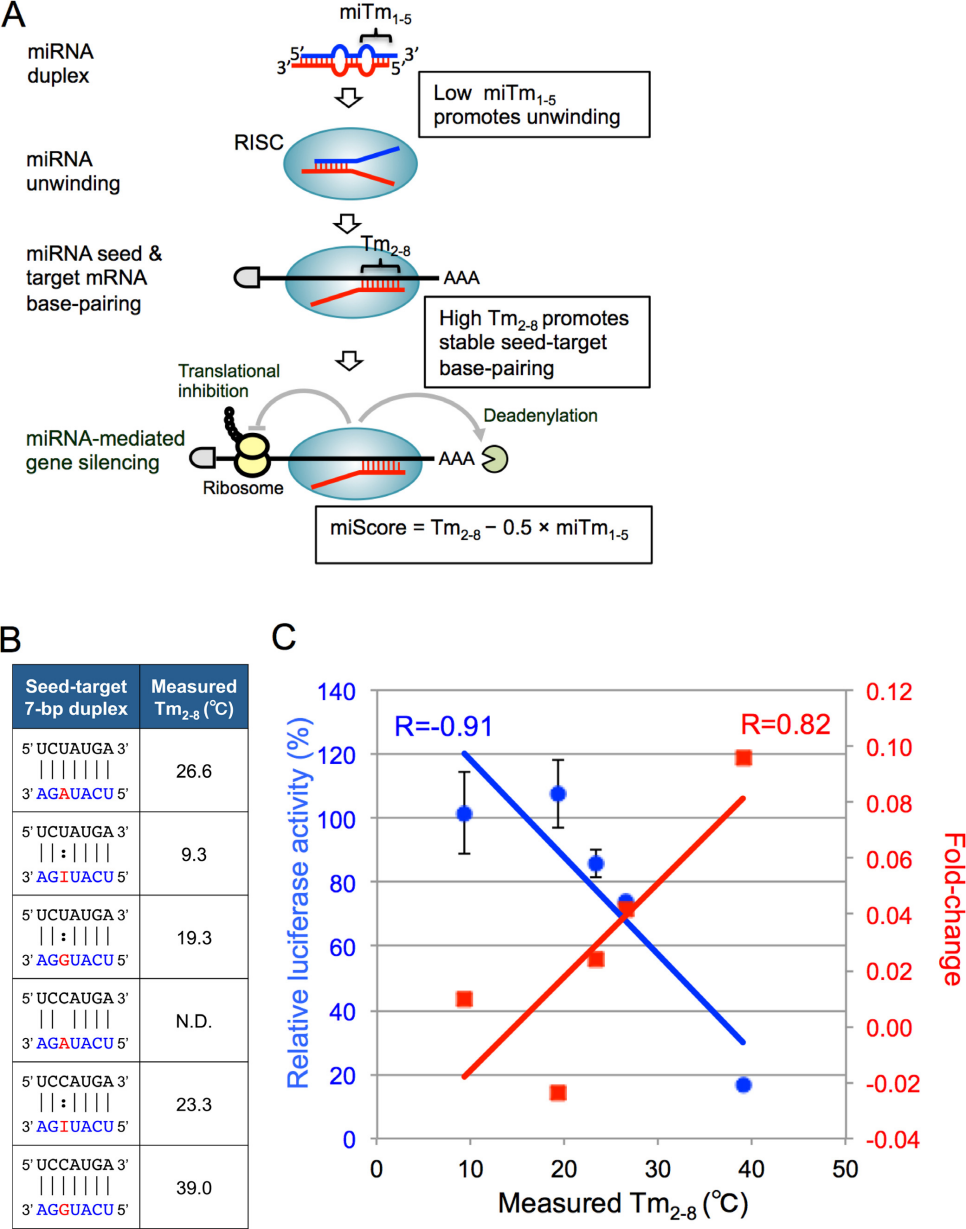
Possible thermodynamic control of miRNA-mediated gene-silencing activity and correlations between Tm values of 7-bp seed-target duplexes and differential fold-changes of expression levels of target transcripts determined by microarray experiments or relative luciferase activities determined by reporter assays. (**A**) The miRNA with low miTm_1–5_ value promotes miRNA unwinding into a single-stranded RNA in the RISC, and that with high Tm_2–8_ value promotes stable base-pairing between miRNA seed region and target mRNA. Thus, the efficacies of miRNA-mediated silencing are determined by the combined thermodynamic parameters that might reflect their unwinding properties (miTm_1–5_) in addition to their base-pairing stabilities in the seed-target duplex (Tm_2–8_) shown as a formula, miScore = Tm_2–8_ − 0.5 x miTm_1–5_. (**B**) The duplex structures formed between 7-mer seed sequence of miR-376a-3p containing adenosine, inosine, or guanosine in the possible editing site and target mRNA sequence with uridine or cytidine at the opposite site of editing position, and the measured Tm values of these 7-bp duplexes. The Tm value of the duplex formed between AGAUACU and UCCAUGA could not be measured, probably due to fairly unstable base-pairing and was shown as not determined (N.D). (**C**) The correlations between the 7-bp Tm values and fold-changes in the expression levels of target mRNAs containing seed complementary sequences in their 3′-UTRs (red), or relative luciferase activities at 50 nM of miRNA duplex (blue). The correlation coefficient (*R*) between Tm values and differential fold-changes was 0.82, and *R* between Tm values and relative luciferase activities was -0.91.

The strand with relatively lower internal stability at the 5′-terminus of the miRNA duplex is preferentially loaded onto the RISC (Figure 7A, 16–18). Furthermore, miRNA canonically recognizes target transcripts using seed-complementary sequences to direct post-transcriptional repression (Figure 7A, 13,14). Consistent with this knowledge, we have published a mathematical model of miRNA base-pairing stability using known thermodynamic parameters of Watson–Crick base-pairing (35,36), and have demonstrated that the silencing efficiency of miRNA is strongly and positively correlated, with the correlation score ‘miScore’ calculated as follows:

\begin{equation*} {\rm miScore} = {\rm Tm}_{2 - 8} - 0.5 \times {\rm miTm}_{1 - 5} \end{equation*}

where Tm_2–8_ means the melting temperature (Tm) value of the seed-target duplex (positions 2–8) and miTm_1–5_ means Tm value of 5′-terminal miRNA duplex (positions 1–5) (Figure 7A). Thus, the seed-target (Tm_2–8_) and 5′-terminal 5-bp duplex (miTm_1–5_) stabilities have opposite effects on gene silencing: miRNAs comprising of an unstable 5′-terminal 5-bp duplex and a stable 7-bp seed-target duplex exhibit strong silencing activity, although the contribution of miTm_1–5_ might be only about half that of Tm_2–8_ because the multiplier coefficient was about 0.5 (12). Therefore, both the difference in thermodynamic stability between the 5′-terminal regions of G-type and I-type miRNA duplexes, and the difference in seed-target binding strength, are likely to contribute to the observed difference in silencing efficiency. Thermodynamic stability in the RNA duplex can be calculated using known parameters (35–37). However, since the thermodynamic parameters of base-pairing between inosine and cytidine have not yet been determined, we annealed both strands of chemically synthesized 5-mer and 7-mer RNA oligonucleotides to form the equivalent of either the 5′-terminal 5-bp duplex and the seed-duplex, respectively, and measured their Tm values using a UV spectrophotometer. In our sequence contexts, sufficient melting curves were obtained only with 7-bp RNA duplexes but not with 5-bp duplexes in 1M NaCl. We selected miR-376a-2 to examine the relationship between silencing efficiency and miScore. Since the editing site is positioned at nucleotide +6 counting from the 5′ end in miR-376a-2, the miTm_1–5_ value of miR-376a-2 is the same for A-type, I-type and G-type miR-376a-2 duplexes, meaning that we can use the Tm_2–8_ value alone instead of miScore. As shown in Figure 7, the measured Tm_2–8_ value for the A-type seed-duplex (formed by the wild-type 7-mer RNA oligonucleotide seed sequence of miR-376a-3p-A and the wild type U-target 7-mer RNA) was 26.6°C, whereas that of the G-type seed-duplex (formed by the C-target 7-mer and the miR-376a-3p-G seed sequence) was the highest at 39.0°C. This is conceivable, as G:C pairs are widely known to be more stable than A:U pairs. Strikingly, the Tm_2–8_ value of the I-type seed-duplex (formed by the C-target 7-mer and the miR-376a-3p-I seed sequence) was 23.3°C, which is low compared to that of the G-type duplex and similar to A:U base-pairing. Furthermore, G:U wobble base-pairing showed even weaker stability at 19.3°C, and I:U pairing showed the lowest Tm_2–8_ value at 9.3°C. The Tm_2–8_ value of the duplex formed between AGAUACU and UCCAUGA (A:C pair) could not be measured, probably due to fairly unstable base-pairing. The Tm_2–8_ values and their silencing activities determined by microarray experiments showed a strong positive correlation (*R* = 0.82). Consistently, the Tm_2–8_ value also showed a strong negative correlation with relative luciferase activity measured at 50 nM of miRNA duplex by reporter assays (*R* = −0.91), suggesting that at least the Tm_2–8_ value behaves like a major determinant for miRNA-mediated gene silencing efficiency for a fixed miTm_1–5_ value. As for miR-22 and miR-191, the editing sites are positioned at +2 and +3 from the 5′, respectively, indicating that the accurate miScores could not be determined, since the miTm_1–5_ values were not successfully measured. In fact, in the case of the miR-22 duplex, the Tm_2–8_ values alone were correlated neither with their silencing activities determined by microarray experiments (*R* = 0.36), nor with luciferase activity (*R* = −0.49) (Supplemental Figure S4). This result suggests that the 5′-terminal 5-bp duplex might also be responsible for miRNA silencing efficacy in the case of inosine-mediated base-pairing. Thus, we propose that inosine in the miRNA 5′-terminal and seed region has the potential to regulate miRNA silencing efficiency by regulating base-pairing stability within RNA duplexes.

The relative variability represented by *R* observed in Figure 7C for the microarray data may be partly due to the different set of non-seed sequences in target mRNAs, which was demonstrated in our and others' previous reports (15,38). Furthermore, in the case of miR-376a-2, although the Tm_2–8_ value of the 7-mer duplex with G:U wobble base-pairing was similar with A:U base-pairing and higher than with I:U pairing, the results of fold-change of microarray experiment as well as relative luciferase activity of reporter assay revealed that G:U base-pairing exhibits no substantial silencing effect (Figure 7), consistent with our previous report (15). This may be caused by the structural perturbation due to quite different glycosidic bond angles formed by G:U wobble base pair compared to Watson–Crick base-pairing (39), which may make it difficult to form stable duplexes between the miRNA seed region and its target mRNAs on the surface of Ago protein as shown in our previous paper (15). Finally, our results have implications beyond the miRNA field, as the thermodynamic profiles of C:G and I:C may also be important for RNA folding, binding affinity with double-stranded RNA-binding proteins, or other base-pairing-associated phenomena.

## CONCLUSION

On the one hand, inosine behaves like guanosine when interpreted by the translational machinery. On the other hand, inosine has different thermodynamic base-pairing characteristics compared to guanosine: guanosine strongly base pairs with cytidine, while inosine base pairs weakly with cytidine. However, the biological significance of this difference in physico-chemical properties had not been well-understood so far. Our microarray analysis reveals that A-to-I editing in the seed region of an miRNA may assign it to different target mRNAs genome-wide. Indeed, wild-type miRNA downregulates target mRNAs with an uridine at the complementary opposite site of editing position, but edited miRNA downregulates target mRNAs with guanosine at that same position. Furthermore, our results effectively demonstrate that the silencing efficiency of miRNAs with inosine differs from that with guanosine, and that this may be due to the difference in base-pairing stability between I:C and G:C pairs. In conclusion, our results unambiguously demonstrate the biological significance of the difference in thermodynamic profiles between inosine and guanosine in the context of RNA duplex formation.

## SUPPLEMENTARY DATA

Supplementary Data are available at NAR Online.

SUPPLEMENTARY DATA
