# Supplementary material for: A-to-I editing in the miRNA seed region regulates target mRNA selection and silencing efficiency
Source: Nucleic Acids Res. 2014 Jul 23;42(15):10050–60. doi: 10.1093/nar/gku662 (PMC4150774; doi:10.1093/nar/gku662)
Supplement: SUPPLEMENTARY DATA [file supp_42_15_10050__index.html]

A-to-I editing in the miRNA seed region regulates target mRNA selection and silencing efficiency — A-to-I editing in the miRNA seed region regulates target mRNA selection and silencing efficiency — A-to-I editing in the miRNA seed region regulates target mRNA selection and silencing efficiency — SUPPLEMENTARY DATA 

# A-to-I editing in the miRNA seed region regulates target mRNA selection and silencing efficiency

## SUPPLEMENTARY DATA

**Files in this Data Supplement:**

- SUPPLEMENTARY DATA
