# Supplementary material for: A-to-I editing in the miRNA seed region regulates target mRNA selection and silencing efficiency
Source: Nucleic Acids Res. 2014 Jul 23;42(15):10050–60. doi: 10.1093/nar/gku662 (PMC4150774; doi:10.1093/nar/gku662)
Supplement: SUPPLEMENTARY DATA [file supp_gku662_nar-01179-y-2014-File003.pdf]

Supplementary Figure S1A: miR−376a−2 duplex (A−type)

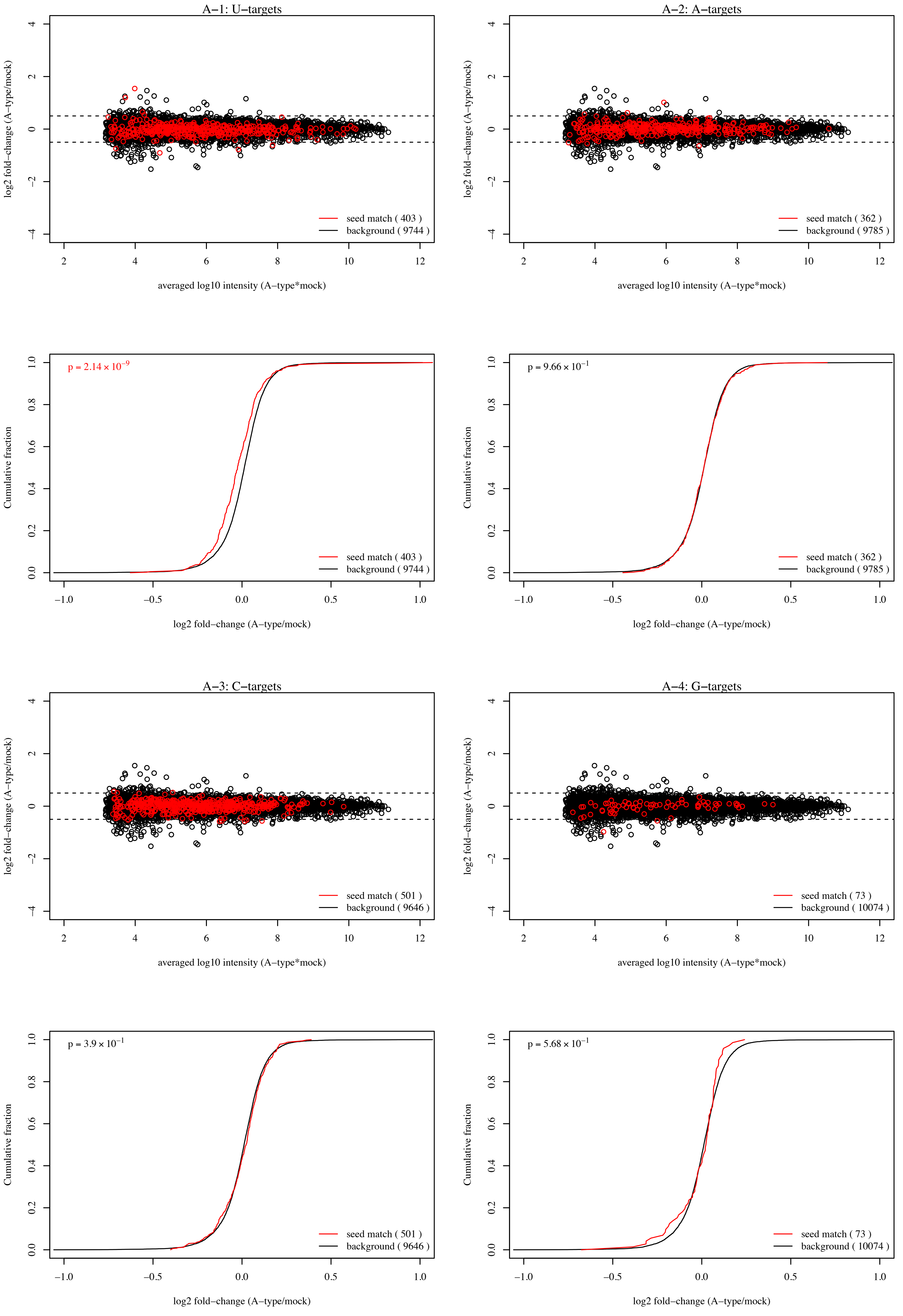

Supplementary Figure S1B: miR−376a−2 duplex (I−type)

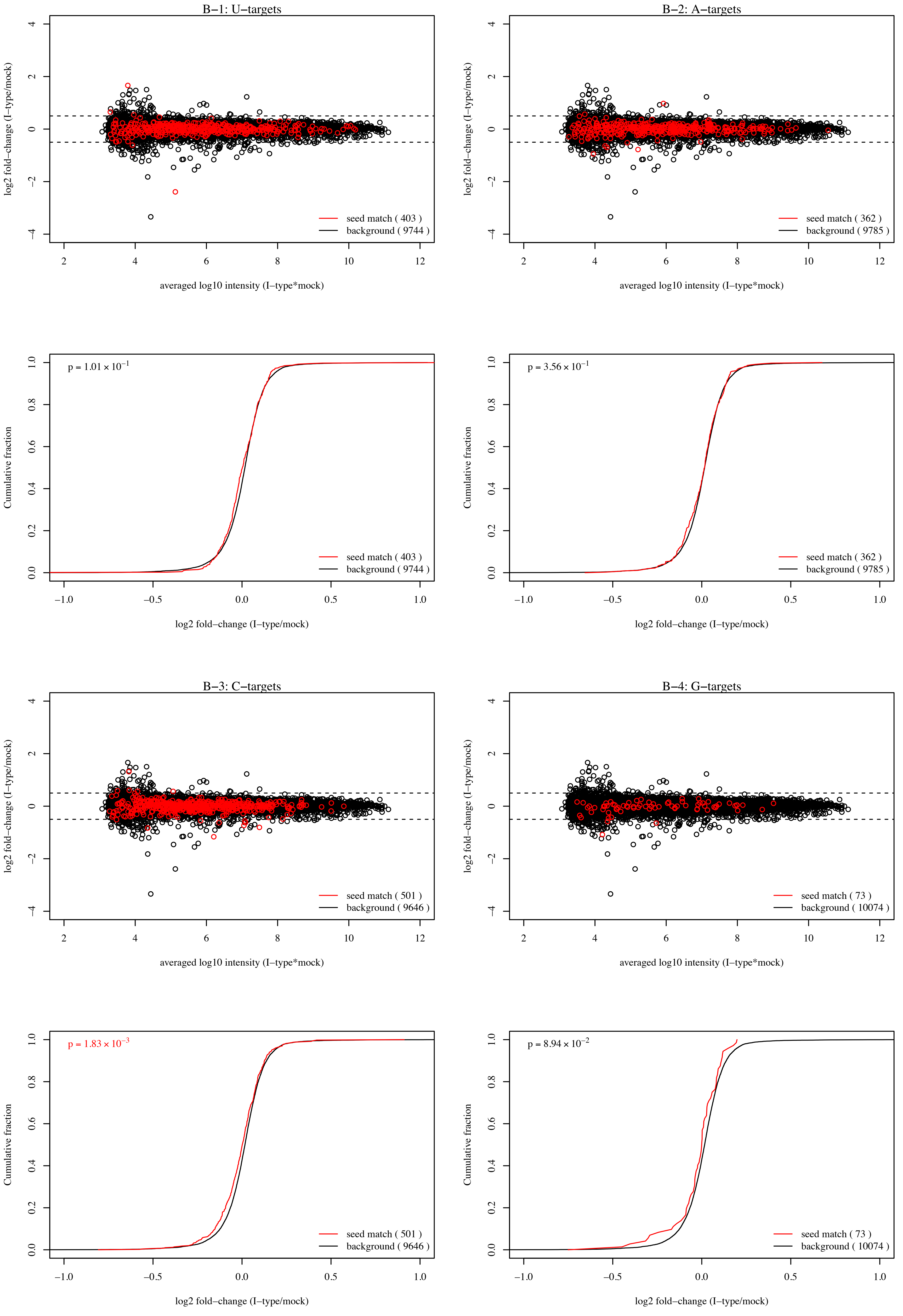

Supplementary Figure S1C: miR−376a−2 duplex (G−type)

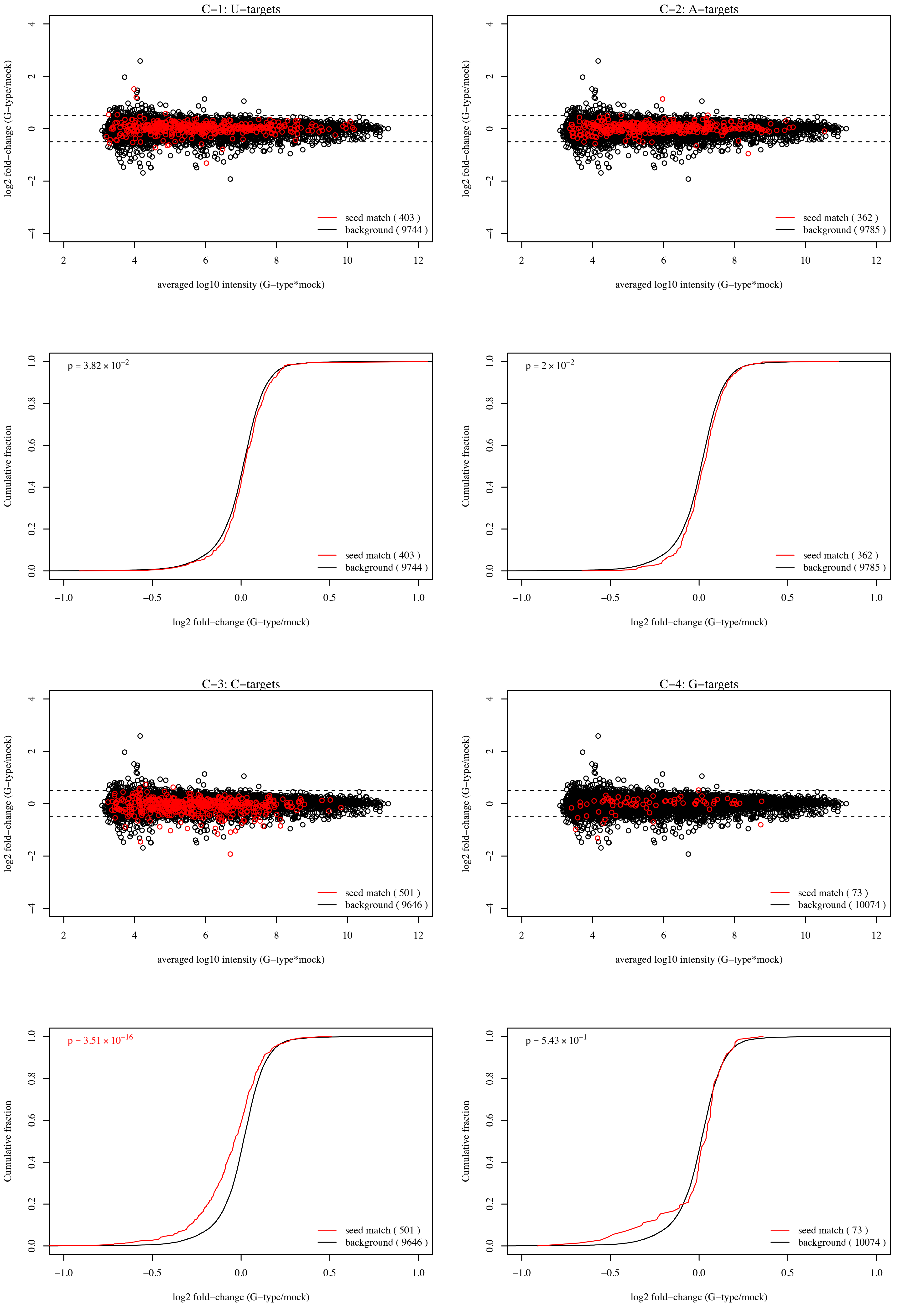

Supplementary Figure S1.

Microarray-based profiling of transcripts downregulated by miR-376a-2 duplexes.

Microarray profiles of the transcripts by the transfection of wild-type (A-type) (A), I-type (B), or G-type (C) miR-376a-2 duplex into HeLa cells 24 hr after transfection. The expression profiles to each of U-targets (A-1, B-1, C-1), A-targets (A-2, B-2, C-2), C-targets (A-3, B-3, C-3), or G-targets (A-4, B-4, C-4) are respectively shown. Upper panels showed MA plots, and lower panels showed cumulative distributions. In MA plot, the vertical axis indicates the changes in gene expression represented by the log2 of fluorescence intensity normalized to that of the–mock transfection sample , and the horizontal axis indicates the log10 of fluorescence intensity multiplied by that of the mock transfection sample. The dotted lines indicate log2(sample/mock) = 0.5 or -0.5. Red dots indicate unique seed-matched target transcripts, black dots, background transcripts. In the cumulative distribution, the vertical axis indicates the cumulative fraction of seed-matched (red) or background (black) transcripts, and the horizontal axis indicates the log2 of fluorescence intensity normalized to that of the mock transfection sample. (A)-(C) The difference in distribution between the seed-matches and their respective backgrounds was estimated using Wilcoxon's rank-sum test. Significant values ($p < 0.01$) are shown in red.

Supplementary Figure S2A: miR−22 duplex (A−type)

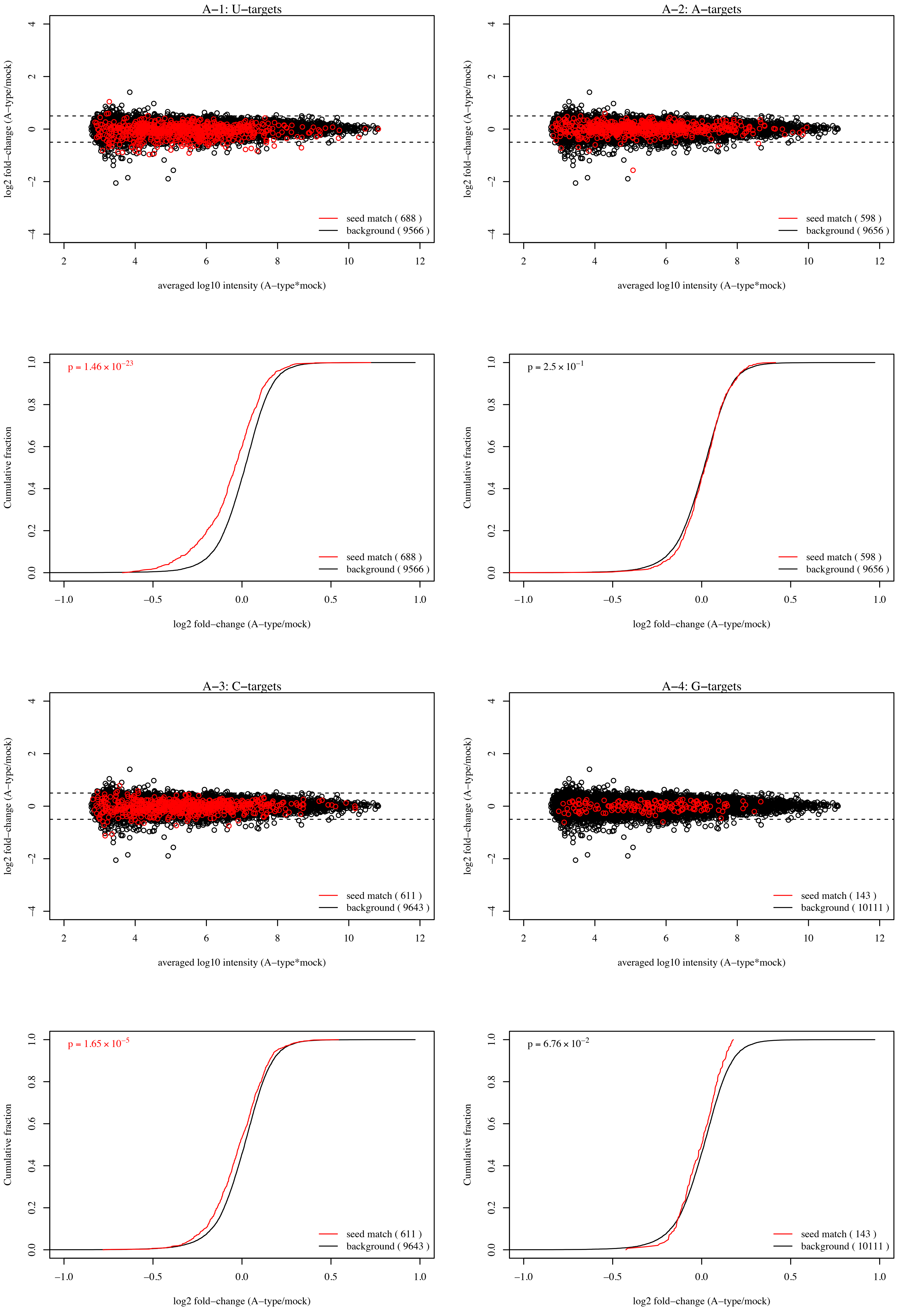

Supplementary Figure S2B: miR−22 duplex (I−type)

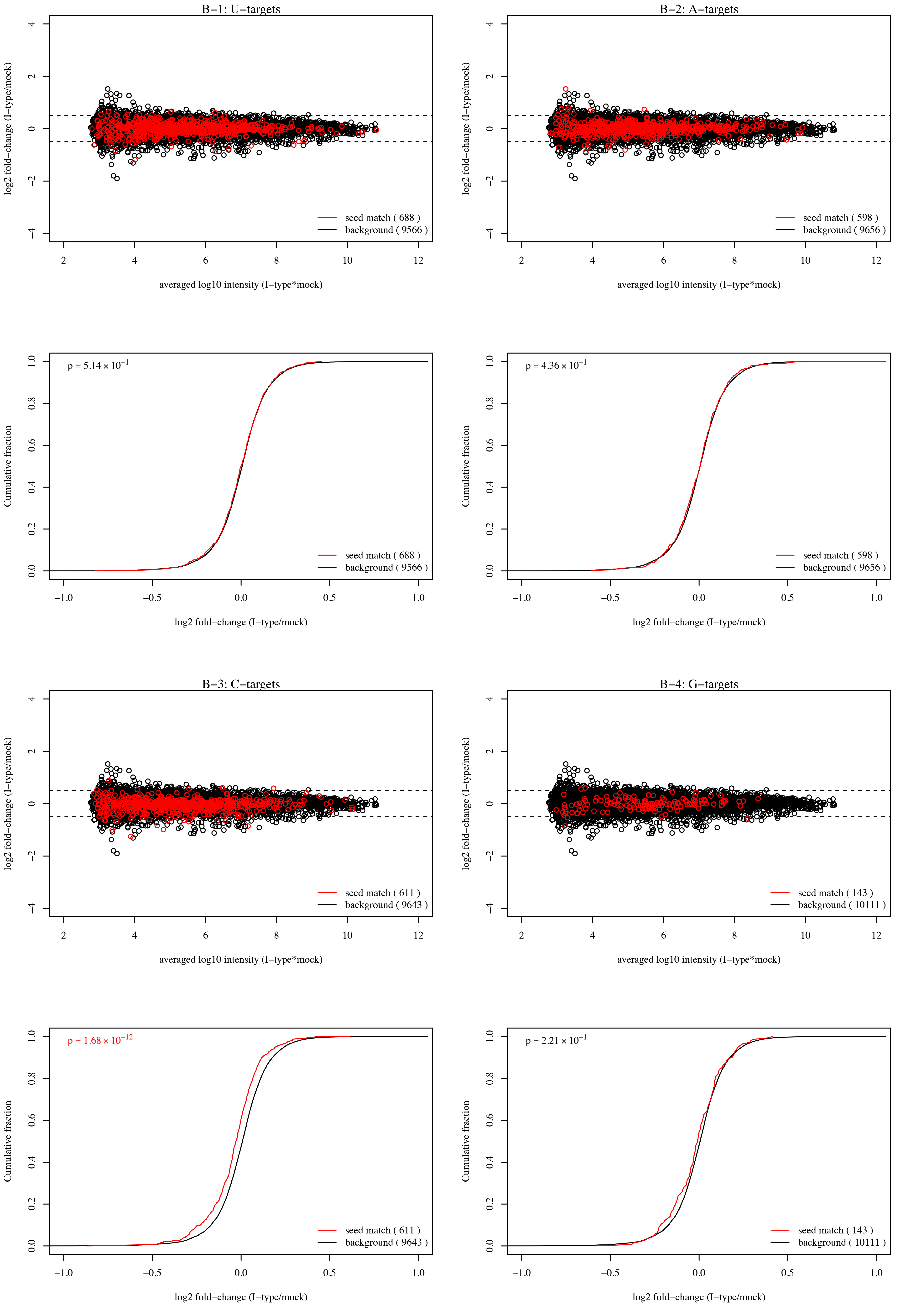

Supplementary Figure S2C: miR−22 duplex (G−type)

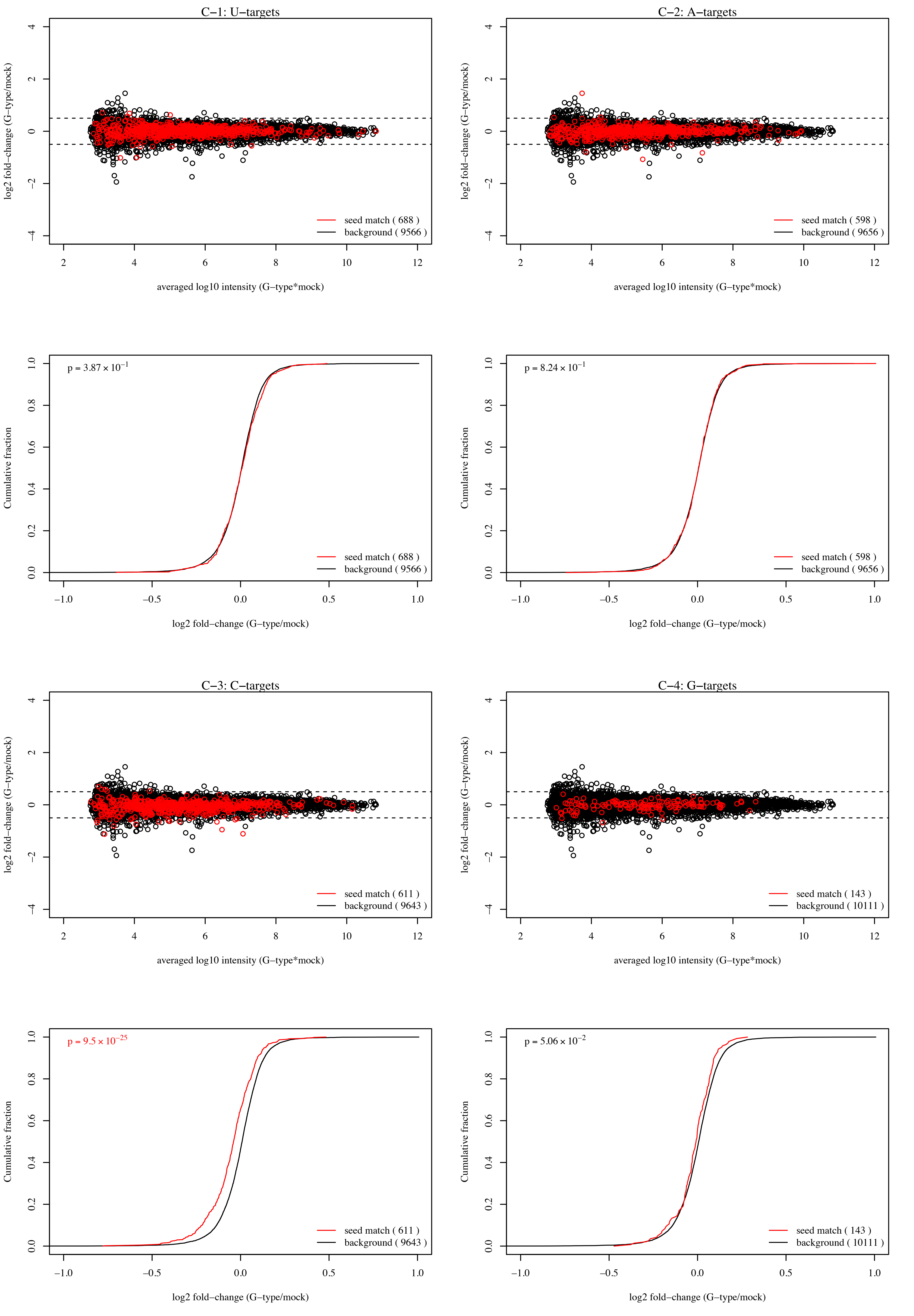

Supplementary Figure S2.

Microarray-based profiling of transcripts downregulated by miR-22 duplexes.

Microarray profiles of the transcripts by the transfection of wild-type (A-type) (A), I-type (B), or G-type (C) miR-22 duplex into HeLa cells 24 hr after transfection. Detailed descriptions about this Figure are the same as in Supplementary Figure S1.

Supplementary Figure S3A: miR−191 duplex (A−type)

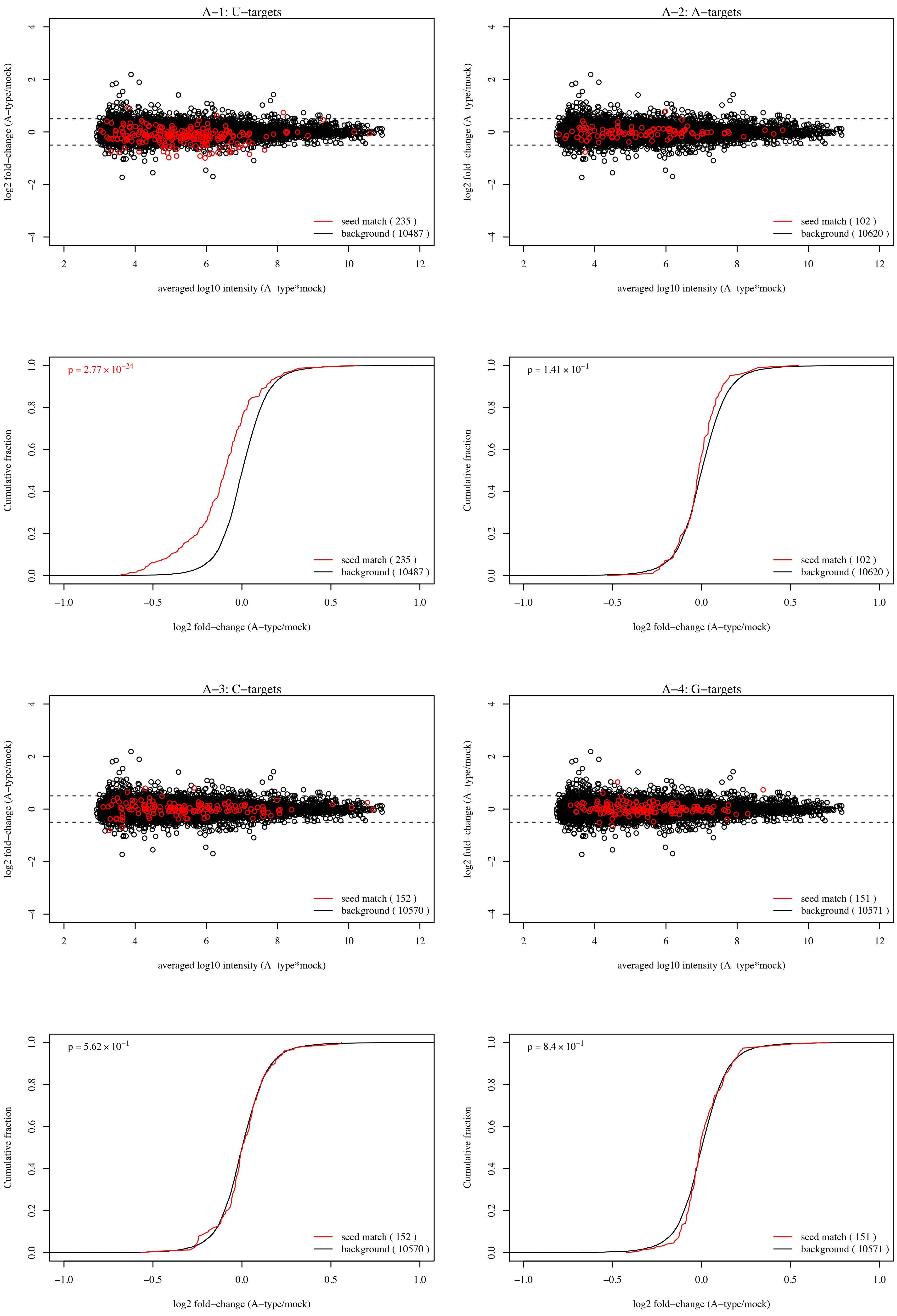

Supplementary Figure S3B: miR−191 duplex (I−type)

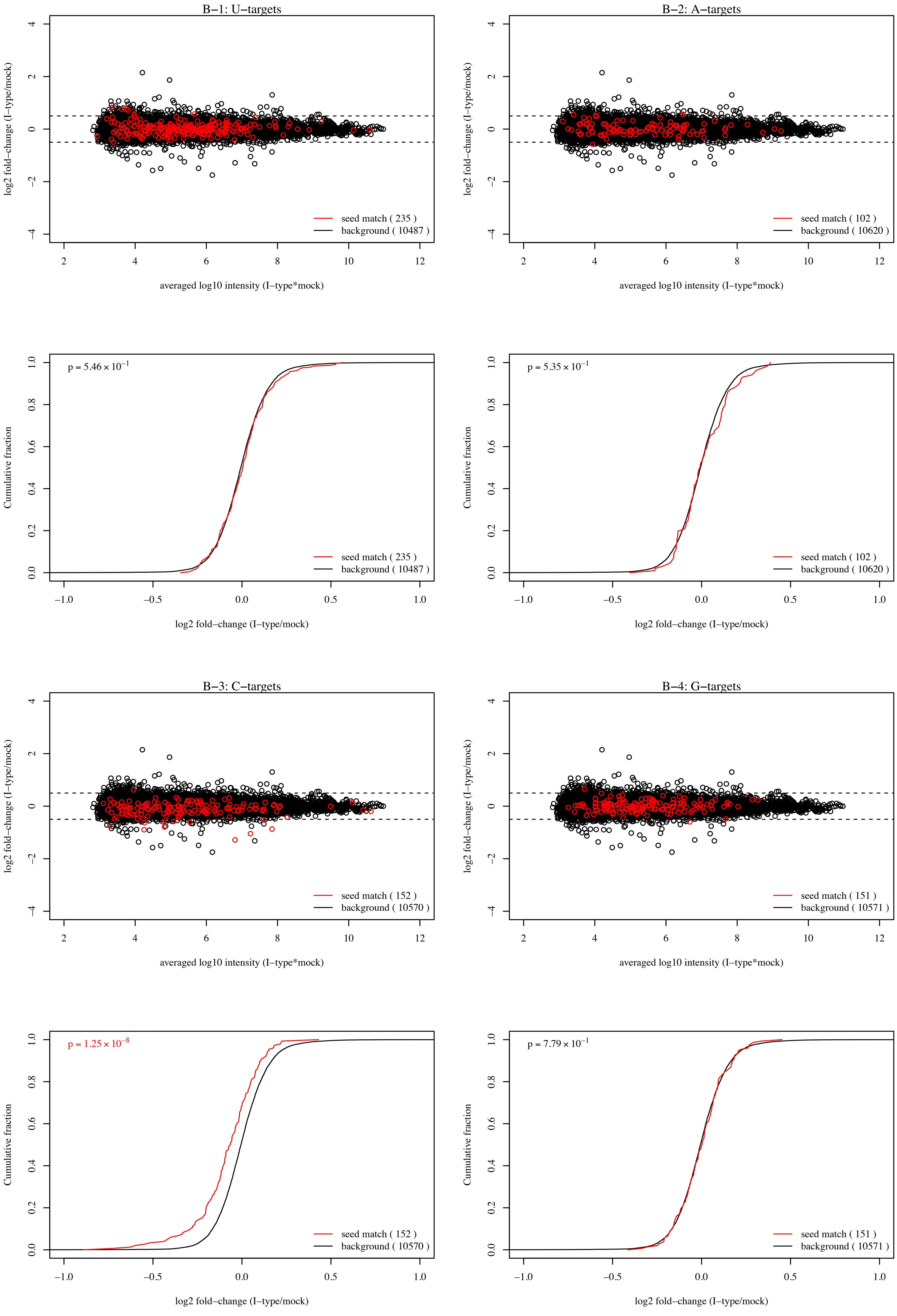

Supplementary Figure S3C: miR−191 duplex (G−type)

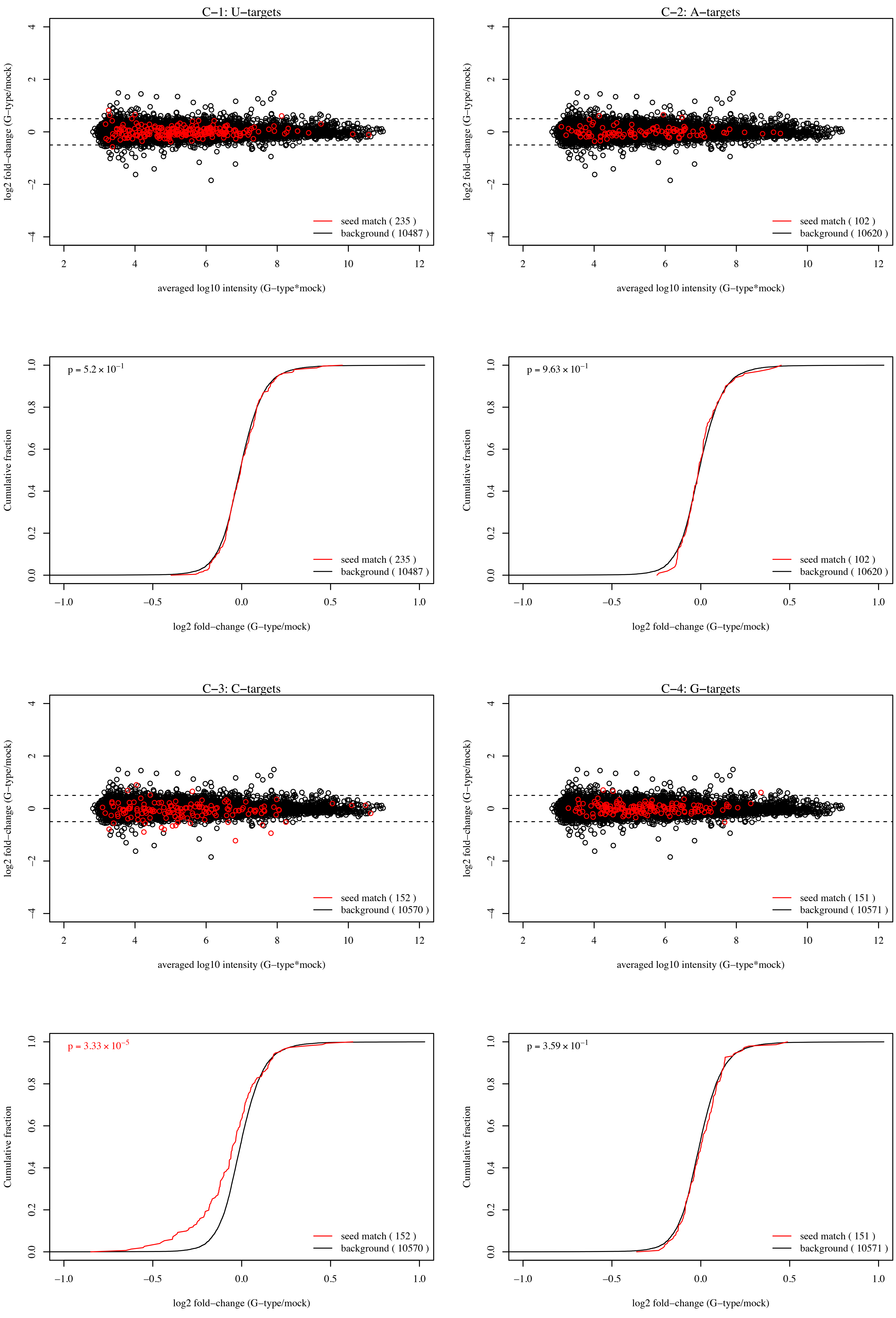

Supplementary Figure S3.
Microarray-based profiling of transcripts downregulated by miR-191 duplexes.
Microarray profiles of the transcripts by the transfection of wild-type (A-type) (A), I-type (B), or G-type (C) miR-191 duplex into HeLa cells 24 hr after transfection. Detailed descriptions about this Figure are the same as in Supplementary Figure S1.

Supplementary Figure. S4.

A

<table>
<tr><th>Seed-target 7-bp duplex</th><th>Measured Tm_{2-8} (℃)</th></tr>
<tr><td>5’ GGCAGCU 3’
|||||||
3’ CCGUCGA 5’</td><td>59.2</td></tr>
<tr><td>5’ GGCAGCU 3’
||||||:
3’ CCGUCGI 5’</td><td>58.3</td></tr>
<tr><td>5’ GGCAGCU 3’
||||||:
3’ CCGUCGG 5’</td><td>59.9</td></tr>
<tr><td>5’ GGCAGCC 3’
||||||
3’ CCGUCGA 5’</td><td>57.9</td></tr>
<tr><td>5’ GGCAGCC 3’
||||||:
3’ CCGUCGI 5’</td><td>59.6</td></tr>
<tr><td>5’ GGCAGCC 3’
|||||||
3’ CCGUCGG 5’</td><td>68.0</td></tr>
</table>

B

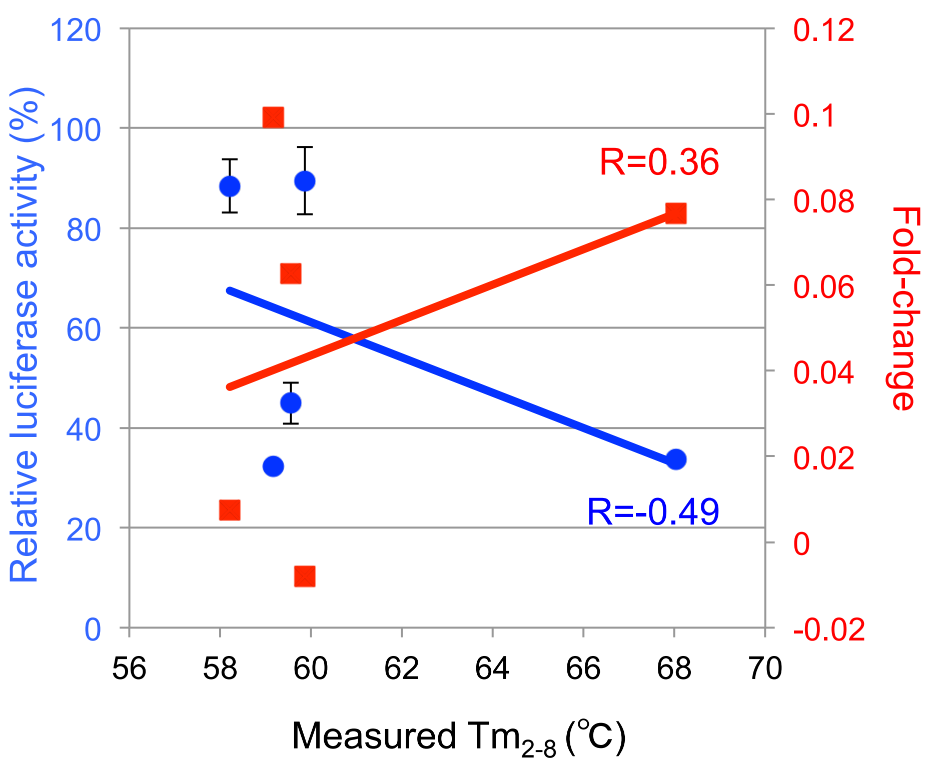

Supplementary Figure S4. Correlations between Tm values of 7-bp seed-target duplexes and differential fold-changes of expression levels of target transcripts determined by microarray experiments or relative luciferase activities determined by reporter assays.

(A) The duplex structures formed between 7-mer seed sequence of miR-22-3p containing adenosine, inosine, or guanosine in the possible editing site and target mRNA sequence with uridine or cytidine at the opposite site of editing position, and the measured Tm values of these 7-bp duplexes.

(B) The correlations between the 7-bp Tm values and fold-changes in the expression levels of target mRNAs containing seed complementary sequences in their 3’-UTRs (red), or relative luciferase activities at 50 nM of miRNA duplex (blue). The correlation coefficient (R) between Tm values and differential fold-changes was 0.36, and R between Tm values and relative luciferase activities was -0.49.

Supplementary Table S1: List of PCR primers

gene name	accession number	Forward primer(5'→3')	Reverse primer(5'→3')
SEL1L3	NM_015187	TGTCTCAGCAAGCGATCCCC	CCACGTGGCTCCGGGTTATT
RAD23A	NM_005053	CACGGAAGCAGCAGGAGAGAACC	CAGGGGGCTCGTTCAGCATCT
CKAP4	NM_006825	GCAGCCACCAGGACTTCTCC	GACTGCACCTTCTGCTCGACG
SLC7A1	NM_003045	AGCGTCCGTTGGTCCTTGAG	GGAAGGTTTCAGAATCCAAGCCG
TTYH3	NM_025250	CTCATCCGCAGCTCCAAGGG	GCAGGATGTCCCCACTCAGC
EPS15	NM_001981	AAAAACGTGGGTTGTATCCCCTGC	TCACGGACCTCCAATCCAGACA

Supplementary Table S2: Inserted oligonucleotide sequences for construction of CM- and SM-targets

Target	Sense strand :inserted oligonucleotide sequence (5'→3')	Antisense strand :inserted oligonucleotide sequence (5'→3')
miR-376a-2-CM-U-target	tcgagACGTGGATTTTCCTCTATGATg	aattcATCATAGAGGAAAATCCACGTc
miR-376a-2-CM-C-target	tcgagACGTGGATTTTCCTCCATGATg	aattcATCATGGAGGAAAATCCACGTc
miR-376a-2-SM-U-target	tcgagTATGCTCGATTAGTCTATGAACCTATGC TCGATTAGTCTATGAACCTATGCTCGATTAG TCTATGAACCg	aattcGGTTCATAGACTAATCGAGCATAGGTTC ATAGACTAATCGAGCATAGGTTCATAGACTA ATCGAGCATAc
miR-376a-2-SM-C-target	tcgagTATGCTCGATTAGTCCATGAACCTATGC TCGATTAGTCCATGAACCTATGCTCGATTAG TCCATGAACCg	aattcGGTTCATGGACTAATCGAGCATAGGTT CATGGACTAATCGAGCATAGGTTCATGGACT AATCGAGCATAc
miR-22-CM-U-target	tcgagACAGTTCTTCAACTGGCAGCTTg	aattcAAGCTGCCAGTTGAAGAACTGTc
miR-22-CM-C-target	tcgagACAGTTCTTCAACTGGCAGCCTg	aattcAGGCTGCCAGTTGAAGAACTGTc
miR-22-SM-U-target	tcgagTATGCTCGATTAG GGCAGCT ACCTATGCTCGATTAG GGCAGCT ACCTATGCTCGATTAG GGCAGCT ACCg	aattcGGTAGCTGCCCTAATCGAGCATAGGTA GCTGCCCTAATCGAGCATAGGTAGCTGCCC TAATCGAGCATAc
miR-22-SM-C-target	tcgagTATGCTCGATTAG GGCAGCC ACCTATGCTCGATTAG GGCAGCC ACCTATGCTCGATTAG GGCAGCC ACCg	aattcGGTGGCTGCCCTAATCGAGCATAGGTG GCTGCCCTAATCGAGCATAGGTGGCTGCCC TAATCGAGCATAc
miR-191-CM-U-target	tcgagCAGCTGCTTTTGGGATTCCGTTGg	aattcCAACGGAATCCCAAAAGCAGCTGc
miR-191-CM-C-target	tcgagCAGCTGCTTTTGGGATTCCGCTGg	aattcCAGCGGAATCCCAAAAGCAGCTGc
miR-191-SM-U-target	tcgagTATGCTCGATTAG TTCCGTT ACCTATGCTCGATTAG TTCCGTT ACCTATGCTCGATTAG TTCCGTT ACCg	aattcGGTAACGGAACTAATCGAGCATAGGTA ACGGAACTAATCGAGCATAGGTAACGGAAC TAATCGAGCATAc
miR-191-SM-C-target	tcgagTATGCTCGATTAG TTCCGCT ACCTATGCTCGATTAG TTCCGCT ACCTATGCTCGATTAG TTCCGCT ACCg	aattcGGTAGCGGAACTAATCGAGCATAGGTA GCGGAACTAATCGAGCATAGGTAGCGGAAC TAATCGAGCATAc
